# Diptheritic Croup and Its Treatment

**Published:** 1872-04

**Authors:** F. W. Bartlett


BUFFALO

Medical and Surgical Journal.

VOL XI. APRIL, 1872. No. 9.

### Original Communications.

ART. I.—*Diphtheritic Croup and its Treatment. A Paper read before the Buffalo Medical Association January 2d,* 1872, *by* F. W. BARTLETT, M. D.

*Mr. President and Gentlemen of the Association:*

In fulfilling a promise, made at our last meeting, to give you the result of my observations upon the nature and treatment of Diphtheritic Croup, I do not expect to instruct my medical friends as regards the nature or symtoms of Diphtheria, or its sequelæ. The disease has occurred so frequently of late years, epidemically or sporadically, that even the recently graduated members of the profession may be supposed to be familiarly acquainted with it. Its treatment, though by no means uniformly successful, and particularly in the croupal complication eminently unsuccessful, has yet come to be practically the same with all regular physicians. The mortality in the cases in which exudation is limited to the faucial region has been greatly reduced, as experience has made us acquainted with the nature of the malady, and it is only in epidemics of extraordinary virulence and severity that the ratio of the deaths to cases is large. But even at this day conflicting ideas prevail. The treatment of the exudation is still subject of discussion. Eminent men hesitate to drop the Probang and Nitrate of Silver, and still apply solutions of 30 to 60 grains to the ounce of water, at short intervals to the inflamed tonsils and fauces. Those less sanguine of the good effects of such treatment, use in a similar manner. Solution of tr. ferri chloride, chlorate potass., alum, chloride of soda, sol. ferri per sulph., tannin and other articles of supposed efficacy. That good is affected by these means I do not deny. The views I hold I shall try to modestly set forth, inviting the friendly criticism of my medical associates.

I start with the conviction that a rude separation of the exuded substance usually covering the tonsilar and faucial region, is always a detriment to the patient. The local treatment, if any is made, should be solvent in its nature, capable of application without violence, and not injurious to the system if it passes to the stomach. It is conceded universally, that the diphtheritic false membrane, plasma, exudation, or by whatever name it may be called is only a local manifestation of a morbid condition of the blood, or the presence in that fluid of specific poison. Whatever its nature, it almost invariably seeks an outlet in the pharynx or parts immediately adjacent.

Has it ever occurred to the gentlemen present to ask the question, why this partiality for the surface mentioned so uniformly appears. Why not appear in the stomach, intestines, lungs, esophagus, conjunctiva, or cutaneous surface, as well as in the parts in the vicinity of the Trachea? I have searched in vain for some explanation of this remarkable fact as regards diphtheria, but nowhere do I find any attempt at a theory; the simple statement seems to satisfy the most inquisitive of our profession. A distinguished medical observer has written that he never saw the exudation in parts removed from atmospheric air. This statement is, I think true, in the case of those who succumbed to the disease in its acute stages. But well attested cases are recorded of diphtheritic heart-clot, exudations, upon the mucous surfaces of the stomach and bowels and other parts reached only by the circulation. Still the main fact is uncontroverted, that in the acute stages the diseased secretion comes by preference to the pharyngeal region. The question as to the period of incubation of diphtheria, is still unsettled. In epidemics of great malignancy, it is probable that the poison soon generates force in the blood, so as to call for a vigorous effort of vitality for its expulsion. But at other times I am of opinion that it may haunt the blood for weeks, and even disappear from the system without any suspicion on the part of the individual that his ill health is due to any such cause; so convinced am I of this, that through the autumn I have given to my patients as a tonic, the tincture of ferri chloride, the symptoms of debility being so similar to what is found in the developed disease. In every case thus treated I have seen excellent results, far superior to those following the use of quinine. Since the great invasion of diphtheria, in 1860 I think, there has been no Autumn or Winter pass without cases of diphtheria of greater or less severity; hence, I infer that it is like the poor, always with us. How far the proving of this as a fact would modify our treatment affords subject for thought. How far the preventive treatment in times of epidemic may avail in individual cases must, of course, largely depend upon its specific effect upon the disease as developed in other subjects. In calling your at ten-tion to a treatment essentially different from that accepted as the best in our times, I am actuated only by a sense of duty to myself, and the profession; ardently desiring to contribute my experiience if of value, and quite as anxious my errors, if such, should be pointed out.

The peculiar treatment I advocate for diphtheritic croup, originated in an accident occurring to one of my patients, and is reported as follows:

In December, 1864, I was called to the lower village of Black Rock, to visit Mr. S. B., a well known citizen. I had no difficulty in diagnosticating Diphtheria of a very malignant form. The tonsils, fauces, posterior nares and epiglottis were thickly covered, with the characteristic exudation. The voice was much altered, the breathing hoarse and croupal, the expression anxious; pulse 120, pain in head severe, debility marked. I sponged the fauces as was the custom, gave quinine, tinc. ferri, chlorate potass., stimulants, beef tea, in short the full and accepted treatment. My patient seemed to do well until the fourth day of attendance; on the evening of that day I found him perceptibly worse. The exudation was pultaceous, of a dark gray color. The pulse 130, weak and irregular. Expression very anxious, and the weakness so great that he did not leave his bed as on other days. I increased his stimulants, urged more beef essence upon him, and was about leaving when he begged that something be given for pain in the epigastric region. For this I recommended a flannel wrung from hot water and lightly sprinkled with spirits terebinthinæ.

Later in the evening Mrs. B. proceeded to carry out my instructions, but as often happens to overworked and weary nurses, forgot and substituted hot turpentine for hot water. This was applied over the entire abdomen. In a few moments pain being insupportable, the patient desired its removal but was told my orders were peremptory and it must remain. It was borne a short time longer and then thrown violently across the room by the patient with the ejaculation " that he would as soon die from the disease as the remedy." Mrs. B. examined and found as she expressed it the whole surface as red as "raw beef." Greatly alarmed she at once covered the surface with soft linen saturated in oil, and impatiently waited my morning visit.

As I entered the sick room I was cheered by the improvement in my patient's voice and appearance, but was soon told by his wife of her sad mistake. On removing the covering from the abdomen I was astonished at the sight that met my view. A surface at least ten by twelve inches in dimension was covered with a thick creamy exudation. The pulse meanwhile had dropped to 90. Inspection showed the intense congestion of the fauces greatly diminished, and the exudation had disappeared from more than half the surface previously occupied. To sum up my patient made a rapid and complete recovery, the denuded surface healing in a few days.

This circumstance made a deep impression upon my mind. and I resolved that in the first great emergency I would put that or a similar plan in practice. Several years elapsed before I had a case I thought urgent enough to demand such treatment. However in September, 1868 one occurred which seemed to justify it.

The patient, a boy of three years, a child of Mr. R. then living on Elk street near the bridge, had been treated for several days by an excellent medical friend who kindly attended my patients at a time of great affliction in my family. An older child had died the week before of so-called pseudo membranous croup, and the patient was under the usual treatment for that affection. On examination I felt satisfied the case was one of diptheritic croup which had progressed to a stage when the most active treatment was required to give any prospect of success. A few moments consultation and the treatment in the case was discontinued, and the usual tonic treatment for diptheria substituted with the following local treatment: A blister size of a silver half dollar was at once applied to the nucha, a tin tube ten inches in length and one-third of an inch in diameter, similar to the blow-gun in use by boys, was procured from a neighboring tin-shop and the fourth part of a teaspoonful of common salt dried and powdered was forcibly blown into the fauces. The disturbance was less than I had ventured to hope. There was scarcely any cough, a little clearing of the throat and expulsion of several teaspoonfuls of yellowish mucus was the only excitement produced. In four hours I called again and repeated the application with similar results and with apparent relief to the breathing which had been performed with great difficulty. Direction was then given to the mother to apply from one fourth to one-third of a teaspoonful every three hours till my next visit. Twelve hours had elapsed when I called again to find the blister formed and commencing to discharge. The respiration had improved, medicine and nourishment had been well borne. The case progressed most favorably, the blister discharging freely and giving but little discomfort to the child. On the sixth day a second blister was applied over the epigastrium which simply furnished a dry red surface, no exudation appearing. By the 12th day the croupal sound had disappeared and the voice was quite restored and ultimately permanently so.

In August of the present year I was called late at night to the house of Mr. M., Pratt street, cor. Sycamore, to prescribe for a child of three years, suffering with what I found to be diptheritic croup. Three days had elapsed without treatment, except a little syrup of Ipecac at intervals. The child was put upon the same treatment as the case just recited and in a few days recovered.

September 30th was called to attend a boy of 13 months, son of Mr. G. D., North Division street, near Hickory. Disease at once recognized as diptheretic croup and same treatment adopted. The parents being informed of the gravity of the mala ly, I was requested to take counsel and invited Dr. S. F. Mixer. That gentleman agreed with me as to diagnosis, and predicted a speedy fatal issue. At his suggestion quinine in two grain doses every third or fourth hour was added to the treatment, but unfortunately, the remedy was almost invariably vomited. Notwithstanding this the patient rapidly improved in respiration, and the blister upon the nucha in 48 hours discharged freely. On the fifth day of treatment the breathing was natural and the voice nearly so.

The patient insisted on being upon the floor and running about, and was taken into the adjoining room on several occasions. To my great regret in some way the child took cold. The blister ceased to discharge, the congestion and engorgement reappeared in the windpipe, the voice failed, and under these grave circumstances I again obtained the advice of Dr. M. We agreed that the case was hopeless, and the child sunk and died on October 9th, the 10th day of treatment and 12th of his sickness.

November 1.—Was called in consultation with Dr. J. C. Green in the case of the son of Mrs. L., Fulton street, near the bridge, aged about 5 years. The child had been ill some three days and was treated in the usual manner with addition of a vapor filling the room from boiling lime water.

The child was breathing with great difficulty, and had strong disposition to sleep, at which times the breathing could be heard in all parts of the house. In consultation it was agreed to apply the blister and use the salt as in the other cases. The case continued steadily to improve until respiration became perfectly natural and the voice resumed its power and volume. In this case only was the vapor of lime water employed, and of its utility I cannot speak, though upon theory I am opposed to it.

As having some bearing upon the disease we are now considering, I wish to mention an unlooked for result of vaccination in two children of Mrs. L., brothers of James whose case I have just detailed. Eight days prior to his sickness I vaccinated two younger brothers of two and four years respectively. Three days before I saw James, Mrs. L. sent for me to look at their arms upon which a number of pustules appeared. Not only upon the arm but also in the left axilla of the elder boy, and extending some three or four inches downward upon the side was a denuded surface profusely discharging. I was at a loss to account for this until the appearance of diptheria in James made it probable that a like poison affected the younger children. Both have progressed favorably.

December 4th I saw all the children. The denuded surface upon the neck of James was entirely and smoothly healed without a scar. That involved in the younger brother had almost entirely healed, and some half dozen slowly suppurating pustules appeared upon the youngest. The general health of all is excellent.

In this case I have alluded to the vapour of lime water disparagingly. I am satisfied that the vapor of a solution of chloride of sodium, or vinegar and water is quite as useful. I condemn all vapors or applications that tend to excite or irritate the mucous membranes. In the latter days of the treatment I have used as I think, the vapor of tar from hot water with advantage.

November 12.—Was called to attend Nelly N. aged 4 years. Parents reside at 291 Fourteenth street. Ill three days when seen. An uncommonly vigorous child. Put promptly upon the usual general treatment. A blister applied as in the other cases, and salt freely used as before described. The croupal distress was very great, and flannels wrung from hot lard were applied to the chest and frequently renewed. There was but little alleviation of symptoms until the blister commenced discharging, but almost from that hour the improvement was marked. By the sixth day the croupal sound disappeared but the voice was not fully restored until nearly two weeks had elapsed. The amount of discharge from the blistered surface was surprising, presenting the appearance of thick mucilage, and a raised line of demarcation was formed which slowly closed in as the patient improved until it contracted to a point in the centre of the denuded tract and healed without a scar.

November 29.—Was called to visit the son of Mr. P. Ellicott street, aged five years, who had been ill for two days. Found incipient croup and diptheritic fauces. Put him on the treatment as used for the other patients. The blister raised the cuticle well but unfortunately having slipped it denuded a larger surface than I desired. Applied the salted lard in the usual way, but the patient complained so much of the pain that against my conviction and judgment I consented to the substitution of wilted cabbage leaves. A serous exudation was the result, in itself as I reason, of no advantage. I caused the room to be filled with the vapor of vinegar and water which can be most readily done by putting half a pint or more of vinegar with a bucket of water in an ordinary wash boilor, and having a good number of flat-irons kept hot upon the stove, they can be suspended readily one by one from an ordinary stove poker, so as to develop any desired amount of vapor. Under the treatment the patient seemed to do well, but I felt troubled at the failure of the blister to furnish the characteristic discharge. False membranes were from time to time ejected, some portions evidently from the trachea, and one piece, quite two inches in length, bifurcated as if coming from the tracheal and bronchial junction. During the day the patient generally breathed easily, and took medicines and food abundantly, but at night respiration was more difficult. The day preceding his decease and the ninth of the sickness he raised without difficulty quantities of soft yellowish mucilagineous exudations, and was very happy and apparently out of danger. At night croupal symptoms reappeared and difficulty of breathing steadily increased. Strength rapidly declined, and he died at about ten o'clock of the morning of December 8th. The parents kindly permitted a section of the trachea to be made, which was performed by my esteemed friends Doctors Daggett and Folwell. Its mucous surface was covered with soft, thin, semi-organized membrane, easily detached, and exposing a surface which had evidently been in a state of engorgement. I attribute the death of my little patient solely to failure to direct the exudations to the cutaneous surface.

December 26th.—Was called to attend the child of Mrs. S. Charles street. Inspection showed yellowish diptheritic patches upon the fauces and tonsils. Child very hoarse and croupy with purplish congestion of the face.

Treatment the same as of last case only that the salted lard was regularly applied to the surface denuded by the blister. Patient has progressed without halt of any kind steadily to recovery. All difficulty of respiration has disappeared and the voice is quite restored. The blister poured out the thick mucus material which I considered the indispensable condition to give promise of cure in any case.

Could we always control the use of moisture, good results might follow, but I think there is no gain in developing a steam so as to saturate walls, bedding, carpets, &c. We can rarely keep the temperature to the point required, and often neglect converts a warm moist air, particularly in the winter months, into a cold or chilly condition. A child is perhaps more likely to take cold when diphtheritic than when in usual health and the consequences are often disastrous.

It is but fair to say that I have within the year treated other cases than those detailed without resort to blistering. It is mortifying to add that they three in number died. I have never seen a case of diphtheritic croup recover under the usual treatment. I estimate that fifty children have died in this city and vicinity in the last four months of pseudo membranous and diphtheritic croup. It is easy to mistake the latter variety for the former with what misfortune as regards the treatment I need not say. A blister in such a case if of moderate size can do no harm and is valuable as a means of diagnosis.

In the use of a blister some care is necessary. The old plan of keeping up a discharge defeats I think the object in view.

If we accept the opinion held by Chaussier, Gordon, and others that the skin consists but of two parts the Cutis vera and Epidermis or cuticle, we need only raise the cuticle to expose to the action of the air the delicate structure of the Corion or Derma. To apply to this surface unguent Sabinae or other irritant simply causes inflammation and the risk of destruction of the true skin. This I think must have been the error of ancient times and the chief cause of the sloughing said to have been observed.

But is it not true that the irrational general treatment adopted by our predecessors was the chief cause of their ill success. Purging, profuse emesis by Antimony, large doses of Calomel, Bloodletting, the spoliative treatment generally. Is it not possible that in condemning their treatment in *toto* we may deprive ourselves of some useful auxiliary to modern practice? I can assure you gentlemen that no scar has followed the use of a blister in any case treated by me. Diphtheria penetrating a wound involving the deeper tissues is a very different thing from an exudation upon a surface complete in its structure and simply deprived of its usual protection from the air. I have used as an excitant nothing more than finely powdered chloride of sodium in lard. This has not failed in 36 to 40 hour to develope the exudation upon the surface. Once there the simple application of salted lard suffices. The dressing should be changed every 3d or 4th hour and the amount of exudation thrown out is surprising. It has been almost invariably light and creamy in color. In two instances a smart crop of pustules appeared, but in no case has there been disposition to gangrene. For applications to the fauces I use only common salt as nearly anhydrous as possible and powdered thoroughly, of this the 4th part more or less of a level teaspoonful may be used and repeated every 3 or 4 hours, extending the time as the patient improves. A tube from which it may be puffed or blown into the fauces can be readily improvised by rolling up a half sheet of foolscap or a note sheet to the diameter of an ordinary pocket pencil. This I secure with thread at either end taking care to have a distinctive mark so that the end inserted into the patient's mouth need never be incautiously used by the attendant. Putting the requisite quantity in the palm of the left hand it is readily scooped into the end of the tube and the tongue being depressed and the end of the tube so carried as not to spill its contents, is passed well into the fauces and emptied by a smart puff through it.

Should this treatment find favor, a bellows operated by the hand would be preferable. I have rarely found any difficulty so far as the patient is concerned. The salt seems at once to deliquesce, unlike alum and other powders. I attach importance to the use of salt in this way. As a gargle it is far inferior—its chemically combining power having been already greatly reduced. Aside from this I credit it with power as an alterative and antiseptic equal if not superior to any article of the Materia Medica. Since I have adopted it as a remedy in diphtheria I have used no other substance as an application for the throat. Just what efficacy to assign to the constitutional treatment as usually practised is not as yet clear. I have not felt disposed to risk my patient's chances by omitting quinine, iron, alcoholic stimulants, or any form of nourishment. What I desire to state is that this treatment alone did not save a single patient in my practice. By the combination as urged upon your notice I have saved all but two in ten cases, and as I have stated one died from inflammation following cold, and the other from failure to direct the exudation to the surface.

One suggestion more and I have done. It is important not to heal the blister by any application. Let it heal in its own way

Twice I have found my patient's prospects endangered by a cessation of the discharge from attempts at healing the surface.

If the blister prematurely ceases to discharge there is a return of the croupal distress and it is well nigh impossible to excite the surface of the blister without creating inflammation. This coagulates the blood in the vessels of the derma and its usefulness as an eliminating surface is at an end. Another blister must be drawn and the time thus lost may prove fatal to success. The points I claim as peculiar in my treatment are the free use of salt to the fauces to the exclusion of all other applications. The use of blisters to be dressed with salted lard, and the vapor of vinegar and water in preference to any other. Add to this the tonic and sustaining treatment in universal use, and you have my idea of the best, only safe and successful treatment of diphtheritic croup. I am aware that errors are easily made in diagnosis, and certain of my cases may be considered by some to indicate a pseudo membranous rather than diphtheritic condition, but if so the large proportion of cures gives encouragement to try the treatment in the former affection.

Of one thing I feel assurred, namely: that blisters can be safely used, and with my present convictions, I must continue to use them until their inutility has been clearly demonstrated.

If a blister is to be applied it should be at the earliest possible stage of the malady, so as to arrest if practicable the initiatory morbid changes in the mucous membrance of the lanynx and trachea. In later stages of the disease it is far less likely to succeed, and if successful the respiration and voice, particularly, are much more slowly restored.

I trust no one who hears me will consider me as urging this treatment in simple tonsilary or pharangeal diphtheria. Such cases are likely to recover under the usual treatment as stated in the earlier part of this paper. It is to prevent suffocation that I urge the trial of the revulsive and eliminating plan.

